# Using an Electromyography Method While Measuring Oxygen Uptake to Appreciate Physical Exercise Intensity in Adolescent Cyclists: An Analytical Study

**DOI:** 10.3390/medicina57090948

**Published:** 2021-09-08

**Authors:** Ștefan Adrian Martin, Roxana Maria Martin-Hadmaș

**Affiliations:** 1 Department of Physiology, “George Emil Palade” University of Medicine, Pharmacy, Science and Technology from Târgu Mureș, Gheorghe Marinescu 38, 540139 Mureș, Romania; 2 Department of Community Nutrition and Food Safety, “George Emil Palade” University of Medicine, Pharmacy, Science and Technology from Târgu Mureș, Gheorghe Marinescu 38, 540139 Mureș, Romania; roxana.hadmas@umfst.ro

**Keywords:** muscle, ventilatory threshold, physical exercise intensity, VO_2_

## Abstract

*Background and Objectives*: During physical exercise, the electrical signal of the muscle fibers decreases due to repeated muscle contractions held at different intensities. The measured signal is strongly related to the motor unit activation rate, which is dependent on the chemical mediators and the available energy. By reducing the energy availability, adenosine triphosphate (ATP) production will decrease and therefore the muscle fibers activation rate will be negatively affected. Such aspects become important when taking into account that the training intensity for many young athletes is rather controlled by using the heart rate values. Yet, on many occasions, we have seen differences and lack of relationship between the muscle activation rate, the heart rate values and the lactate accumulation. *Materials and Methods*: We conducted a prospective analytical study conducted during a 4-month period, on a sample of 30 participants. All study participants underwent an incremental exercise bike test to measure maximum aerobic capacity as well as the muscle activation rate in the vastus lateralis by using an electromyography method (EMG). *Results*: With age, the EMG signal dropped, as did the electromyography fatigue threshold (EMG_FT_) point, as seen through *p* = 0.0057, r = −0.49, CI95% = −0.73 to −0.16, and electromyography maximum reached point (EMG_MRP_) (*p* = 0.0001, r = −0.64, CI95% = −0.82 to −0.36), whereas power output increased (*p* = 0.0186, r = 0.427). The higher the power output, the lower the signal seen by measuring active tissue EMG_FT_ (*p* = 0.0324, r = −0.39) and EMG_MRP_ (*p* = 0.0272, r = −0.40). Yet, with changes in median power output, the power developed in aerobic (*p* = 0.0087, r = 0.47), mixed (*p* = 0.0288, r = 0.39), anaerobic (*p* = 0.0052, r = 0.49) and anaerobic power (*p* = 0.004, r = 0.50) exercise zones increased. *Conclusions:* There has been reported a relationship between aerobic/anaerobic ventilatory thresholds (VT_1_ and VT_2_) and EMG_FT_, EMG_MRP_, respectively. Each change in oxygen uptake increased the power output in EMG_FT_ and EMG_MRP_, improving performances and therefore overlapping with both ventilatory thresholds.

## 1. Introduction

Performance during physical exercise is related to various systems and their function within the human body. As a result, the number of physiological tests has increased steadily for the better assignment of daily physical activity for both amateur and elite athletes [1,2,3].

Previously, each testing period started with anthropometric measurements as well as speed, resistance and general strength, while oxygen consumption, cell metabolism, movement economy, strength, power and the type of muscle fiber were one by one included in athletic training [4]. By applying various physiological tests, one can obtain information concerning some variables that are used to guide daily physical training activity, such as the heart rate, the power output and the oxygen saturation, as well as lactate values, which are the most commonly used [5,6,7]. However, the same existing data have been reanalyzed before, during and after physical training to achieve a better understanding of the physiological state. Similar objectives have been previously stated when using data regarding neuromuscular resistance, which was studied by using the electromyography (EMG) method to improve both physical strength and endurance [8]. However, it seems that the muscle activation rate, strongly related to exercise intensity and exercise performance [9] is more used nowadays. However, during physical exercise, the electrical signal of the muscle fibers decreases due to repeated muscle contractions held at different intensities. More specifically, the measured signal is strongly related to the motor unit activation rate, which is dependent on the chemical mediators and the available energy. By increasing the current injections above the alpha-motor neuron threshold, the firing frequency will decrease and the power output will change due to lower excitability [10]. Yet, the mechanism is much more complex. According to several papers, the main changes are strongly related to the physical exercise intensity, while the genetically acquired nerve and muscle structures ensure a different response during physical exercise [11,12].

The muscle fibers response depends on factors that influence a neuromuscular excitability [13]. The main ones are related to the existing slow, fast or mixed muscle fibers that affect the physiological response during physical exercise through certain cellular structures [14]. Low muscle fibers have higher oxidative capacity, unlike fast muscle fibers, which have a fast oxidative and a fast glycolytic metabolism that affect resistance during physical exercise [14]. By further studying the energy metabolism, the signaling of muscle fibers can be related to both pulmonary ventilation and oxygen uptake, which can therefore increase the use of adenosine triphosphate (ATP) and the muscle fiber activation rate [15]. However, there is less available information regarding both cell metabolism and pulmonary adaptations, as well as the muscle activation rate following each exercise intensity zone in adolescent athletes. Such aspects become important when taking into account the fact that the training intensity for many young athletes is controlled by using heart rate values. Yet, on many occasions, we have seen differences and lack of relationship between the muscle activation rate, the heart rate values and the lactate accumulation rate. As a result, we thus believe that by reducing the energy availability, ATP production will decrease [16] and therefore the muscle fibers activation rate will be negatively affected. Based on our hypothesis, similar outcomes should be observed in adolescent groups but differences may exist due changes in cell metabolism during low to moderate physical exercise. Therefore, through this paper we also want to approach the relationship between energy metabolism, minute ventilations and muscle activation rate to improve physical training guidance.

## 2. Materials and Methods

We conducted a prospective analytical study over a 4-month period, between February and May 2021. All procedures described in the study methodology were performed in the Advanced Medical and Pharmaceutical Research Center (CCAMF) of “George Emil Palade” University of Medicine, Pharmacy, Science and Technology of Târgu Mureș, Romania.

Prior the participants’ inclusion stage, the authors obtained the approval of the Ethical Committee (259/14.11.2018). The study research, through the study methodology, complied with the Helsinki Declaration regarding Experimental Procedures and Human Research.

### 2.1. Study Participants

Prior the inclusion, all research procedures were explained to the participants and their caregiver/s. After their acceptance, the inclusion in the study sample was completed.

The study sample consisted of 30 participants who were recruited freely, through an announcement send to the local road cycling, cross country mountain bike or triathlon sports clubs. To be included in the study sample, the participants had to meet the following inclusion criteria: (1) report to the selection stage as scheduled; (2) age between 14 and 17 years old; (3) inclusion as a junior athlete in one of the following sports: road cycling, cross country mountain bike or triathlon; (4) no acute or (5) chronic pathologies that could have interacted with one or more of the research procedures. If any of the inclusion criteria were not met, the participant/s were excluded from the study sample.

### 2.2. Test Applied

All study participants underwent an incremental exercise bike test to measure maximum aerobic capacity as well as the muscle activation rate in the vastus lateralis by using an EMG method.

### 2.3. Anthropometric Measurements

Prior the incremental exercise test, all participants underwent anthropometric measurements on one occasion during which the body weight (kg) and the body height (cm) were measured in minimal clothing by using a calibrated thaliometer (ADE Germany GmbH, Hamburg, Germany). The HARPENDEN Professional Skinfold equipment (Baty International Ltd., Burgess Hill, UK) was used to measure the biceps, the triceps, the subscapular, the suprailiac, the abdominal, the thigh and the leg skin folds. All measurement results, as well as age, gender, body weight and body height results were applied in the Durnin and Womersley formula [17] to obtain information regarding the fat mass and fat-free mass, expressed as kilograms (kg) or percentage (%) of the body weight. On each occasion, the results were also reported as height for age and weight for age percentiles (%).

### 2.4. Measuring the Maximum Rate of Oxygen Consumption during the Incremental Exercise Test

The maximum oxygen consumption (VO_2max_) was measured by applying the Bruce Maximal Testing Protocol conducted on the General Electric eBike (General Electric, Chicago, IL, USA) while using the Cortex Metalyzer 3B equipment (Cortex Medical, Leipzig, Germany) for gas exchange analysis. Before each test or when the equipment was requested, we calibrated the equipment with known O_2_ and CO_2_ concentrations. The turbine was also calibrated before each test with air volumes that were individually adapted to the sex- and age-related normal values.

During the test, the participants had to maintain 75 revolutions per minute (rpm) until exhaustion in one stage of the incremental exercise test. The test consisted of 10 (*n* = 10) intensity stages, each of 3 min length, during which the physical exercise intensity was measured in watts (W). The first stage started from 50 W resistance and then in each step the resistance was increased by 25 W. The test was completed when the respiratory exchange ratio (RER) was equal or above 1.10, if the predicted maximum heart rate (HR_max_) was reached, oxygen consumption (O_2_) capped, with values not exceeding 150 mL or if the individuals reached voluntary exhaustion and failed to maintain 75 revolutions per minute. By using the measured data, we further determined the ventilatory threshold 1 (VT_1_) and the ventilatory threshold 2 (VT_2_) by applying the V Slope method [18]. Other parameters were measured, of which we mention the tidal volume (VT L/min), as well as respiratory frequency (Rf, c/min), oxygen consumption (VO_2_, L/min or mL/min/kg), respiratory exchange ratio (RER) and oxygen pulse (VO_2_/HR, b/mL).

### 2.5. Measuring the Electromyography Fatigue Threshold during Incremental Exercise Test

The electromyography fatigue threshold (EMG_FT_) and the electromyography maximum reached point (EMG_MRP_) were measured during the Incremental Exercise Test and therefore during the maximum rate of oxygen consumption measuring period. The electrical signal was measured by using the Biopac MP36 R system (BIOPAC, Goleta, CA, USA). The leg was measured between the anterior superior spina iliaca and the lateral part of the patella in order to place the electrodes on the vastus lateralis. By confirming the placement location, the hair was removed from the electrode placement area and the surface was cleaned by using alcohol and sterile materials [19,20,21]. The electrodes were placed 20 mm apart. The participants were asked to step on the bike in order to adjust the cycling position by taking into account the body height; the connection cables were placed to check the signal. The signal was filtered by using a band-pass filter of 5–500 Hz with a channel-sampling rate of 200.00 Hz, while also measuring the EMG Root Mean Square at a similar sampling rate of 200.000 Hz.

The EMG_FT_ was determined by processing all data. By using measurements from each stage, average values were assigned for every 20 s of the incremental exercise test. Therefore, for each stage of the maximum rate of oxygen consumption test held during the incremental exercise test, the time and the EMG amplitude were interpolated to confirm whether the regression line was significant (*p* < 0.05). The highest power output, without significant difference (*p* > 0.05), was set as the EMG_FT_ point. The EMG_MRP_ was determined as the signal measured during the pre-exhaustion stage. All measurements were determined in microvolts (µV) and millivolts (mV) unit measures.

### 2.6. Statistical Evaluation

The statistical evaluation was carried out with the GraphPad Prism 6.0 software (Graph Pad Software, San Diego, CA, USA), with a level of significance set at α = 0.05. The tests used for the inferential assessment were: the D’Agostino and Pearson omnibus normality test for data distribution; and the Mann–Whitney test for proving the differences between two items and the Spearman r test for assessing the relationship between two analyzed parameters. The data were presented by using descriptive data such as the median value, the minimum/maximum values and the variation coefficient (CV).

## 3. Results

The median age was 17 (14 to 23) years old. The median body weight was 61.8 kg (47.6 to 78.5), and the body height was 170 cm (156 to 181). We measured 12.66% median fat mass (6.08 to 26.07) and 54.91% fat-free mass (43.04 to 71.52).

### 3.1. Maximum Rate of Oxygen Consumption during the Incremental Exercise Test

During the VO_2max_ test, the absolute power output was 3.7 W/kg. However, the average power output during the three-minute stages completed before exhaustion was 3.41 W/kg. In addition to these data, VO_2_ reached 2.78 L/min absolute oxygen consumption and 47 mL/min/kg relative oxygen consumption, with 124 L/min VE due to 2.52 L/min tidal volume, 55 c/min respiratory frequency and 1.25 RER maximum reach. The median oxygen consumption at VT_1_ was 1.88 L/min, while at VT_2_, oxygen consumption was 2.5 L/min. VT_1_ value was equivalent to 66.15% of the maximum oxygen uptake, whereas VT_2_ was 89.13% of the maximum oxygen uptake. Both VT_1_ and VT_2_ were used to determine the exercise zones by using O_2_ uptake and power output, as further detailed in Table 1.

### 3.2. Age and Anthropometric-Induced Changes on the Incremental Exercise Test Results

With age, the EMG signal dropped similar to EMG_FT_ point, as seen through *p* = 0.0057, r = 0.49, CI95% = −0.73 to −0.16 and EMG_MRP_ (*p* = 0.0001, r = −0.64, CI95% = −0.82 to −0.36), whereas power output increased (p = 0.0186, r = 0.427). The fat-free mass changed with body weight (*p* = 0.0001, r = 0.871). Furthermore, with changes in fat-free mass, there were changes in the maximum power reach (*p* = 0.0001, r = 0.72, CI95% = 0.47 to 0.86), while oxygen uptake increased (*p* = 0.0001, r = 0.697, CI95% = 0.44 to 0.84) and RER dropped (*p* = 0.0017, r = −0.54, CI95 = −0.763 to −0.22). Overall, VT_1_ was higher with age and body weight (*p* = 0.0005, r = 0.59), similar to VT_2_ (*p* = 0.0001, r = 0.81), which was correlated with EMG_FT_ point (*p* = 0.0013, r = 0.56).

### 3.3. The Electromyography Measure Results during Incremental Exercise Test

The EMG signal was continuously measured during the incremental exercise test. The median EMG_FT_ point was set at 125 W (100 to 175), 2.02 W/kg (1.61 to 2.83), respectively. Equivalent to the power output, we measured an electrical signal of 0.19 mV (0.06 to 0.52 mV) and 185.5 µV (62.59 to 520 µV), 49.28% CV. Therefore, EMG_FT_ was set at 63% (47.2 to 81.47%) of the maximum signal value. We also recorded the EMG_MRP_ point at 200 W (175 to 275), CV 16.91%, 0.26 mV (0.08 to 0.62 mV) and 260.6 µV (82 to 615.8 µV), reached at 93.92% of the maximum signal reach.

### 3.4. Relationship between Oxygen Consumption, the Ventilatory Thresholds and EMG Measures

The maximum power reach increased the median power output during the last stage before exhaustion (*p* = 0.0001, r = 0.914, CI95% = 0.86 to 0.96). Yet, both VT_1_ (*p* = 0.0001, r = 0.720) and VT_2_ (0.0001, r = 0.84) were correlated with the power output in all exercise zones. With oxygen uptake (*p* = 0.0001, r = 0.89) and oxygen pulse (*p* = 0.0001, r = 0.79) the power output increased while lower RER value were measured (*p* = 0.0284, r = −0.40). No relationship was seen between power output and respiratory parameters, specifically VT, RF and VE (*p* > 0.05). The higher the power output, expressed as W/kg, the lower the signal seen by measuring active tissue EMG_FT_ (*p* = 0.0324, r = −0.39) and EMG_MRP_ (*p* = 0.0272, r = −0.40). Yet, with changes in median power output, the power output during aerobic (*p* = 0.0087, r = 0.47), mixed (*p* = 0.0288, r = 0.39), anaerobic (*p* = 0.0052, r = 0.49) and anaerobic power (*p* = 0.004, r = 0.50) exercise zones increased. Similar changes were seen in EMG_MRP_ power output, which increased (*p* = 0.0007, r = 0.58) as against EMG_FT_ (*p* > 0.05), which failed to change as related to the power output during the last incremental stage before exhaustion. However, the absolute power output changed again only with EMG_MRP_ (*p* = 0.0265) unlike EMG_FT_ (*p* > 0.05). With maximum oxygen uptake, both VT_1_ (*p* = 0.0001, r = 0.81) and VT_2_ changed (*p* = 0.0001, r = 0.93), as they were also related to both VT (*p* = 0.0269, r = 0.40) and VE (*p* = 0.018, r = 0.42). VO_2_ value increased power output during aerobic (*p* = 0.0001), mixt (*p* = 0.0001), anaerobic (*p* = 0.0001) and anaerobic power (*p* = 0.0001) exercise zones.

Each change in oxygen uptake increased power output at EMG_FT_ (*p* = 0.0001, r = 0.73) and EMG_MRP_ (*p* = 0.0001, r = 0.842) without any correlations with the electrical signal intensity in the EMG_FT_ and EMG_MRP_ stages (*p* > 0.05). VO_2_/HR increased with VT (*p* = 0.0214, r= 0.41) and VE (*p* = 0.0251, r = 0.40) while lower RER values were measured (*p* = 0.0302, r = −0.39). Overall, VO_2_/HR increased VT_1_ (*p* = 0.0001, r = 0.86) and VT_2_ (*p* = 0.0001, r = 0.88), the power output during all exercise zones (*p* < 0.05) as well as the power output in EMG_FT_ (*p* = 0.0001, r = 0.827) and EMG_MRP_ (*p* = 0.0001, r = 0.80) without relating to the intensity of the electrical signal (*p* > 0.05). Further data are illustrated in Table 2.

**Figure 1:**
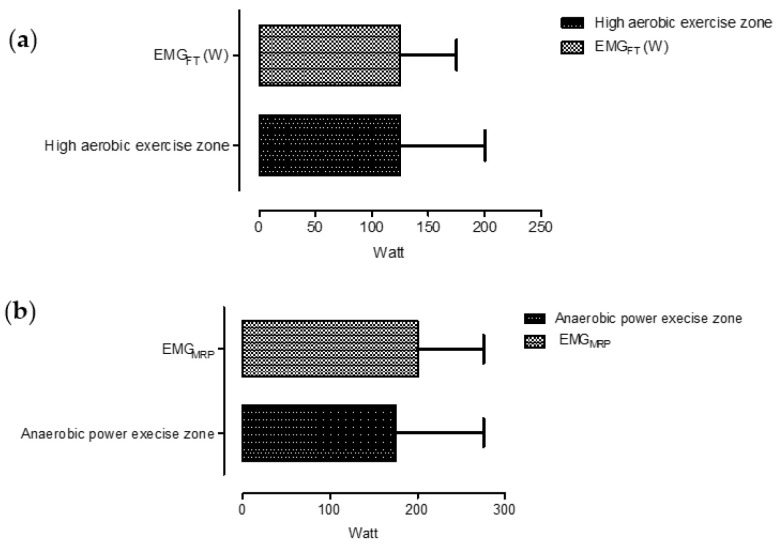
Illustration of the power output during high aerobic exercise zone, the fatigue threshold EMG_FT_ point (**a**) and the electromyography maximum reached point (EMG_MRP_) (**b**), presented as median value and range.

VT_1_ was correlated to both EMG_FT_ (*p* = 0.0011, r = 0.56) and EMG_MRP_ (*p* = 0.0001, r = 0.81), similar to VT_2_ which was correlated to EMG_FT_ (*p* = 0.0001, r = 0.73) and EMG_MRP_ (*p* = 0.0001, r = 0.83). However, the mixed exercise zone was strongly correlated to EMG_FT_ point (*p* = 0.0001, r = 0.647), showing us that the highest power output before supplying energy under anaerobic conditions overlaps with EMG_FT_, as further detailed in Figure 1.

## 4. Discussion

We studied the relationship between the ventilatory thresholds, minute ventilation, oxygen uptake and EMG signaling in the vastus lateralis during an incremental exercise test. According to the study outcome, EMG_FT_ was correlated to the power output during VT_1_, while VT_2_ power output was correlated to EMG_MRP_. Yet, oxygen consumption increased the power output for all physical exercise zones while the electrical signal for EMG_FT_ and EMG_MRP_ failed to change.

### 4.1. Relationship between Power Output, Oxygen Consumption and Muscle Activation

During early research, authors confirmed the importance of using the EMG_FT_ point in both recreational and professional athletes to assess exercise intensity and fatigue [21]. However, up to this point, few papers completely suggested the replacement of the means of measuring and determining the ventilatory thresholds by using the EMG method [22].

As such, the current research paper used oxygen consumption as the main indicator of aerobic performance, similar to Bearden et al. [23]. However, Tikkanen et al. [24] used blood lactate to study physical exercise intensity. In our study, changes in oxygen uptake easily influenced power output and therefore performance, but changes in power output did not necessarily mean improvements in oxygen uptake. We failed to measure lactate accumulation during the incremental exercise test, but by matching several papers one can observe with training, changes in lactate tolerance and performance improvements without major changes in oxygen uptake [25]. Yet, all changes are related to oxygen consumption, distribution and cell-level use, which, according to Wagner et al. [26] is much more related to the cell structure and the main adaptations within muscle, respiratory and cardiovascular systems. According to Jabbour et al. [27], physical exercise is related to both mechanical work and efficiency, which is further influenced by various physiological systems. Based on Enoka et al.’s published paper, the mechanical work is dependent on the nervous system, which will send signals to activate the motor units (UM) and contract the muscles [28]. As such, mechanical work is influenced by neural connections, nerve, muscle structures along with energy metabolism, and therefore energy systems that hence changes according to EMG amplitude, as seen in our paper. As to aerobic physical exercise, several differences are observed in the cellular structures of the human body. The neuromuscular junction seems to be different in slow versus fast muscle fibers [29]. In fast muscle fibers, which are more likely used during long but high intensity physical exercise, such as cross-country skiing, running or mountain biking, a larger junction is seen. With a higher junction in fast muscle fibers, several authors described a higher safety factor as to lower muscle fibers [30]. Likewise, fast muscle fibers have a higher postsynaptic membrane with a high number of acetylcholine (ACh) receptors and therefore a higher number of sodium channels that will increase current density [31] and improve power output over EMG_FT._ However, according to Lermakers et al. [13], there is an important limitation regarding the firing rate by reducing membrane excitability in fast muscle fibers. In contrast, slow muscle fibers have the opposite structure and therefore a lower safety factor that activates slow muscle fibers during low to moderate physical exercise intensity and during physical exercise, which uses external dynamic resistance. We also believe that these adaptive structures and mechanisms improve resistance during physical exercise.

Based on our outcomes, we can easily understand that the testing protocol influences individual response during physical exercise. This is why a relatively small number of papers applied a three-minute protocol stage. However, during both short and long stages, amplitude differences can and will occur due to exercise intensity [19]. Of the main influencing factors, one can observe both local and central factors which influence the activation of both type I and type II muscle fibers [32] that can explain the main changes within our study group. For example, according to Wan et al. [11] the EMG_FT_ point is corresponding to the activation of a large number of oxidative-glycolytic muscle fibers, while the EMG_MRP_ is further related to the activation of a large number of glycolytic muscle fibers. This can also be the main reason for the association with ventilatory thresholds, considering the increase in CO_2_ simultaneously with the activation of fast muscle fibers according to exercise intensity. Exercise intensity will much more induce changes in EMG amplitude, which can be used to determine both EMG_FT_ and EMG_MRP_. Following the relationship between VT_1_ and EMG_FT_, one can relate the intramuscular rise in [H^+^], as seen in Tikkanen et al.’s paper [24], which confirms the sensitivity to muscle acidosis. Chemical changes within the blood as to the main chemo and baroreceptors [33], induce signals that are sent to increase minute ventilation, which can further be used to determine the ventilatory threshold. As such, a higher number of muscle fibers are recruited and therefore the signal amplitude changes, similar to our outcome. According to Enoka et al., the main mechanism by which a larger number of motor units are used refer to the motor unit recruitment and to the rate of coding, which is strongly related to the muscle group and the force levels [28]. Yet, there is a possibility that EMG_FT_ is reached earlier than VT_1_. Such outcomes are specific during low neuromuscular resistance, which means that the current method can also be used to characterize exercise intensity and the potential effect it has on the muscle system, unlike Mohamed et al. who failed to obtain a relationship between EMG and muscle length during physical exercise [34]. Therefore, we believe that similar evaluations could offer information regarding neuromuscular resistance and therefore individual performance, which is not related to any other factor, as seen in Harrison et al.’s [35] paper.

### 4.2. Study Limitations

The current research has various limitations. Thus, the study sample is restricted and therefore further research is needed to draw conclusions that are more accurate. Furthermore, several muscle groups should be studied at the same time, which we failed to manage due to technological limitations. In addition to oxygen consumption, lactate production should be measured as well during the incremental exercise test.

## 5. Conclusions

There is a relationship between the EMG signal, the power development and the age of the participants. With age, the EMG signal dropped in EMG_FT_ and EMG_MRP_ points, while the power output and the oxygen uptake increased as the respiratory exchange ratio dropped. By following the results, the power output mainly increases with oxygen uptake and oxygen pulse, while no relationship is seen with individual performance VT, Rf and VE. Yet, a relationship has been reported between both VT_1_/ VT_2_ and EMG_FT_/EMG_MRP_, respectively. Each change in oxygen uptake increased the power output in EMG_FT_ and EMG_MRP_, improving performances and therefore overlapping with both VT_1_ and VT_2_. However, further research should aim to study the effect that physical exercise held above EMG_FT_ and EMG_MRP_ has on muscle endurance and therefore performance.

## Figures and Tables

**Table 1:** Power Output for each exercise zone illustrated as median values.

Exercise Zone	Median Power Output, Watt	CV, %	% Of Max Value	CV, %
Aerobic exercise zone	100 (30 to 160)	26.59	50.86 (36 to 58.15)	13.92
Mixed exercise zone	125 (95 to 200)	22.58	68 (54.29 to 82.67)	12.62
Anaerobic exercise zone	155 (114 to 245)	21.55	81.14 (65.14 to 92.67)	9.73
Anaerobic power exercise zone	175 (141 to 275)	19.41	Maximum reach	

Legend: CV = Coefficient of variation, % Of Max Value = percentage of the maximum reached value.

**Table 2:** Comparative data over the cardiopulmonary test results and EMG measures.

STUDY PARAMETERS	VT_1_ 1.88, 1.26 to 2.9 L/min		VT_2_ 2.5, 1.76 to 3.6 L/min		VO_2_ 2.78, 2.38 to 3.89 L/min		EMG_FT_ 125, 100 to 175 W		EMG_MRP_ 200, 175 to 275 W	
	*p*	r	*p*	r	*p*	r	*p*	r	*p*	r
**Body weight**, 61.8, 47.6 to 78.5 kg	0.0005	0.59	0.0001	0.27	0.0001	0.69	0.0001	0.78	0.0001	0.65
**Fat-free mass**, 54.91, 43.04 to 71.52 kg	0.0001	0.66	0.0001	0.85	0.0001	0.82	0.0001	0.80	0.0001	0.71
**Power reach before exhaustion**, 3.41, 2.61 to 4.4 W/Kg	0.0175	0.43	0.066	0.33	0.0001	0.83	0.042	−0.37	0.0007	0.58
**Absolute power reach**, 3.7, 2.98 to 4.8 W/Kg	0.0553	0.35	0.345	0.17	0.0001	0.84	0.885	−0.02	0.026	0.40
**Power output in *low aerobic exercise zone***, 100, 63 to 160 W	0.0001	0.79	0.0001	0.88	0.0001	0.84	0.0001	0.69	0.0001	0.89
**Power output in *high aerobic exercise zone***, 125, 95 to 200 W	0.0001	0.74	0.0001	0.89	0.0001	0.82	0.0001	0.64	0.0001	0.83
**Power output in *anaerobic exercise zone***, 155, 114 to 245 W	0.0001	0.78	0.0001	0.87	0.0001	0.84	0.0001	0.64	0.0001	0.83
**Power output in *anaerobic power exercise zone***, 175, 141 to 275 W	0.0001	0.82	0.0001	0.86	0.0001	0.84	0.0001	0.72	0.0001	0.91

Legend: VT_1_ = ventilatory threshold 1, VT_2_ = ventilatory threshold 2, VO_2_ = oxygen consumption, EMG_FT_ = electromyography fatigue threshold, EMG_MRP_ = electromyography maximum reaching point, *p* = probability level, r = Pearson product-moment correlation coefficient, CI95% = confidence interval of 95%.
